# Network meta-analysis and cost per responder of targeted Immunomodulators in the treatment of active psoriatic arthritis

**DOI:** 10.1186/s41927-018-0011-1

**Published:** 2018-02-12

**Authors:** Vibeke Strand, M. Elaine Husni, Keith A. Betts, Yan Song, Rakesh Singh, Jenny Griffith, Marci Beppu, Jing Zhao, Arijit Ganguli

**Affiliations:** 1 Stanford University, Palo Alto, CA USA; 2 Cleveland Clinic, Cleveland, OH USA; 3 Analysis Group Inc., 333 South Hope Street, 27th Floor, Los Angeles, CA 90071 USA; 4 Analysis Group Inc., Boston, MA USA; 5 AbbVie Inc., Mettawa, IL USA

**Keywords:** Arthritis, psoriatic, Meta-analysis, Immunomodulators, Cost-benefit analysis

## Abstract

**Background:**

Multiple targeted immunomodulators (TIMs) for psoriatic arthritis (PsA) treatment are available, but limited studies have directly compared these agents. This study indirectly compared the efficacy of TNF-α, interleukins, and phosphodiesterase-4 inhibitors for treatment of active PsA.

**Methods:**

A systematic literature review was conducted to identify phase III randomized controlled trials (RCTs) for adalimumab, certolizumab pegol, etanercept, golimumab, infliximab, ustekinumab, secukinumab, and apremilast in active PsA. Joint (ACR20/50/70) and skin outcomes (PASI75/90) at Week 24 with each TIM were estimated via a Bayesian network meta-analysis, and the incremental cost per responder over the first 24 weeks of treatment was calculated. Similar analyses were conducted in a subgroup of biologic-naïve patients.

**Results:**

Seventeen RCTs were identified; 13 included ACR and/or PASI responses at Week 24. Among the overall population, patients receiving adalimumab, golimumab, and infliximab showed higher ACR20/50/70 (adalimumab: 61.2/42.8/40.8%, golimumab: 61.6/39.8/27.4%, infliximab: 56.2/57.1/34.2%) and PASI75/90 (72.7/55.5%, 74.1/57.2%, and 77.1/61.0%, respectively) responses at Week 24 compared with other TIMs. In terms of cost-effectiveness, these treatments were also associated with the lowest incremental cost per responder for both skin and joint outcomes. Similar rankings of efficacy and incremental cost per responder were observed in the analysis among biologic-naive patients.

**Conclusions:**

Adalimumab, golimumab, and infliximab were associated with higher efficacy and lower incremental costs per responder for both joint and skin responses in active PsA.

**Electronic supplementary material:**

The online version of this article (10.1186/s41927-018-0011-1) contains supplementary material, which is available to authorized users.

## Background

Psoriatic arthritis (PsA) is a chronic inflammatory arthritis that occurs in up to 24% of psoriasis patients [1]. In the majority of PsA patients, skin symptoms precede the arthritis, and common manifestations of the disease may include synovitis, enthesitis, dactylitis, and anterior uveitis [2]. Similar to rheumatoid arthritis, PsA can be disabling and lead to erosive arthropathy in some patients [3]. A variety of therapeutic agents are available for the management of PsA, although the clinical heterogeneity of the disease poses a challenge to clinicians in determining the best treatment [4]. PsA patients with moderate to severe symptoms typically require disease modifying anti-rheumatic drugs (DMARDs), non-steroidal anti-inflammatory drugs (NSAIDs), phototherapy, or a combination of the three [5]. Recent studies have shown that traditional DMARDs, such as methotrexate, are ineffective for preventing progression of joint damage and may have serious adverse effects [6–8].

Immunomodulators (TIMs) such as tumor necrosis factor-α (TNF) inhibitors, interleukin inhibitors, and phosphodiesterase-4 inhibitors have dramatically changed the therapeutic paradigm of PsA [9]. Although demonstrated to be more effective than traditional DMARDs, the relative efficacy and cost-effectiveness of these newly investigated agents remains uncertain. Reliable evidence regarding the comparative efficacy of these novel PsA agents is crucial for informing clinical and economic decisions about their most appropriate use.

The objective of this study was to determine the comparative efficacy of these TIMs, as well as the incremental cost per responder over the first 24 weeks of treatment in patients with active PsA in the US. Since few head-to-head studies of these therapies have been performed, a network meta-analysis was conducted to synthesize all available evidence from randomized trials and enable indirect comparisons among competing interventions [10]. Detailed methodological reviews and implementation guidelines for network meta-analyses have been published [11, 12], and network meta-analyses have become a preferred source of evidence among researchers, medical decision makers, and health technology assessment agencies [12–14].

## Methods

### Trial identification

A systematic literature review was conducted to identify Phase III randomized controlled trials (RCTs) of TNF inhibitors (adalimumab, certolizumab pegol, etanercept, golimumab, and infliximab), interleukin inhibitors (secukinumab and ustekinumab), and a phosphodiesterase-4 inhibitor (apremilast) for active PsA. Trials were conducted in adult patients (age ≥ 18) with active PsA, and included one of the TIMs listed above as active treatment versus placebo or versus another active comparator. RCTs were required to have reported clinical outcome measures for joint responses (by American College of Radiology [ACR] criteria) and/or skin responses (by Psoriasis Area and Severity Index [PASI]). ACR criteria is commonly used to assess the improvement in tender or swollen joint counts, acute phase reactant, patient and physician global assessments, pain scale, and disability/functionality questionnaire. PASI score evaluates the effectiveness of treatment through the assessment of psoriasis lesions in four body regions: head, upper extremities, trunk, and lower extremities. For inclusion, treatment arms were required to use the dose approved by the US Food and Drug Administration (FDA) for each TIM.

### Efficacy measures

Joint responses were defined by the ACR20 (20% improvement in ACR criteria), ACR50, and ACR70 response criteria [15]. PASI75 (75% improvement in PASI) and PASI90 responses were used to define skin responses [16, 17]. Efficacy measures at Week 24 were used in the current study, as they were the primary outcome measures for newly investigated agents, including ustekinumab and secukinumab.

### Costs

Unit drug costs in the US as of May 8, 2017 were based on wholesale acquisition costs (WAC) obtained from ReadyPrice®. Dosing schedules for each TIM were based on FDA labeling. Costs for infliximab were based on treatment costs of an 80-kg adult and administration costs (intravenous infusion) as of May 8, 2017 were obtained from the US Department of Health and Human Services (CPT code 96413 for the initial hour and 96415 for the subsequent 3 h) [18, 19]. Costs for each TIM over the first 24 weeks of treatment were calculated based on dosing schedules, acquisition costs, and infusion costs.

### Statistical methods

A Bayesian network meta-analysis was conducted to assess the comparative efficacy (in terms of ACR20/50/70 and PASI75/90) of different TIMs in the treatment of active PsA. Using a fixed effect model, the number of patients achieving ACR20, ACR50, and ACR70 at Week 24 was assumed to follow a binomial distribution, with the corresponding probabilities linked to the treatment effects via a *logit* function. Non-informative priors were applied to the treatment effect parameter to ensure that treatment comparisons were driven by the observed data. Estimated ACR responses were summarized using posterior means and 95% credible intervals (CrI) for all treatments included in the network.

For the PASI outcomes, a fixed effect, ordinal model assumed that the number of patients achieving PASI50, PASI75, and PASI90 responses followed a multinomial distribution. A *probit* link was used to estimate the probability of each treatment achieving PASI responses based on all observed comparisons. This model allowed the three PASI outcomes to be analyzed jointly, and further assumed that each treatment had the same magnitude of additive effect versus placebo on subsequent levels of PASI responses on the inverse probit scale [20]. A non-informative prior was also specified for the response rates of the reference arm across all RCTs. Based on the model results, PASI75 and PASI90 responses for each therapy were estimated.

Numbers needed to treat (NNT) for each additional responder were calculated as the reciprocal of the difference in estimated response rate between active agent and placebo based on the network meta-analysis. Incremental cost per responder for each treatment relative to placebo was calculated as the product of the total costs over the first 24 weeks of treatment and the NNT. All analyses were conducted within a Bayesian framework and were estimated via Markov chain Monte Carlo using OpenBUGS 3.2.3. Analyses were repeated in the subset of patients without prior biologic treatment.

## Results

**Fig. 1:**
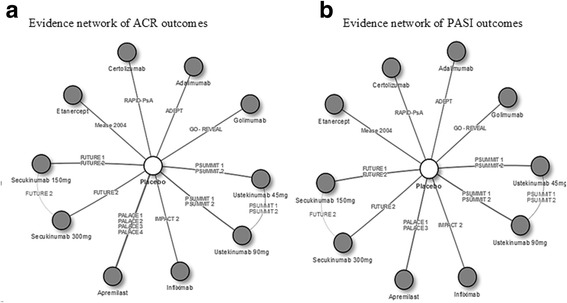
Evidence network for ACR and PASI outcomes among the overall population. **a** Thirteen trials reported ACR responses at Week 24 were selected: ADEPT [34], PALACE 1 [39], PALACE 2 [40], PALACE 3 [41–43], PALACE 4 [44, 45], RAPID-PsA [46], Mease 2004 [25], GO-REVEAL [47], IMPACT 2 [48], FUTURE 1 [49], FUTURE 2 [50], PSUMMIT 1 [51], and PSUMMIT 2 [52]. (**b**) Eleven trials reported PASI responses at Week 24 were selected: ADEPT [34], PALACE 1 [39], PALACE 3 [41–43], RAPID-PsA [46], Mease 2004 [25], GO-REVEAL [47], IMPACT 2 [48], FUTURE 1 [49], FUTURE 2 [50], PSUMMIT 1 [51], and PSUMMIT 2 [52].

The systematic literature review identified 17 RCTs that met the inclusion criteria. One trial [21] was excluded because it was only 12 weeks in duration and three other trials [22–24] were excluded because placebo patients crossed over to active treatment prior to Week 24. The remaining 13 trials reported ACR and/or PASI responses at Week 24 after initiation of treatment (Additional file 1: Table S1). Proportion of patients with conventional DMARDs use and with methotrexate (MTX) use at baseline was summarized. Evidence networks of ACR and PASI outcomes among the overall population are shown in Fig. 1a and b, respectively.

Across the selected RCTs, 11 provided stratified results for biologic-naive patients or were conducted in a biologic-naive population (Additional file 2: Table S2). Although Mease 2004 [25] did not explicitly indicate biologic treatment experience of participants, they were assumed to be all biologic-naïve given the era in which this trial was conducted. PASI responses in biologic-naive PsA patients were not available for apremilast and certolizumab pegol. Evidence networks of ACR and PASI outcomes among the biologic-naïve population are shown in Additional file 3: Figure S1A and S1B, respectively.

### Network meta-analysis: ACR outcomes

Among the overall population, three TNF inhibitors -- golimumab, adalimumab, and infliximab -- demonstrated better ACR outcomes compared with other TIMs at Week 24. PsA patients treated with golimumab had the highest ACR20 responses (61.6%), followed by adalimumab (61.2%) and infliximab (56.2%). In terms of ACR50, infliximab had the highest efficacy (57.1%), followed by etanercept (46.6%), adalimumab (42.8%), and golimumab (39.8%). In terms of ACR70, adalimumab (40.8%), infliximab (34.2%), and golimumab (27.4%) had higher efficacy compared with other TIMs (Table 1).

**Table 1:** ACR response rates and NNT at Week 24 among the overall population

Treatment	ACR20		ACR50		ACR70	
	Response rate (95% CrI)	NNT (95% CrI)	Response rate (95% CrI)	NNT (95% CrI)	Response rate (95% CrI)	NNT (95% CrI)
Placebo	17.0% (15.4%, 18.7%)	–	7.0% (5.9%, 8.2%)	–	2.5% (1.9%, 3.3%)	–
Adalimumab	61.2% (47.8%, 73.6%)	2.3 (1.8, 3.2)	42.8% (27.0%, 62.5%)	2.8 (1.8, 4.9)	40.8% (15.9%, 82.2%)	2.6 (1.3, 7.4)
Apremilast	33.4% (27.1%, 40.4%)	6.1 (4.4, 9.5)	15.5% (10.9%, 21.8%)	11.8 (7.0, 23.5)	5.0% (2.7%, 9.1%)	40.3 (15.8, 222.7)
Certolizumab pegol	50.2% (38.6%, 62.3%)	3.0 (2.2, 4.6)	27.9% (18.0%, 41.8%)	4.8 (2.9, 8.9)	16.7% (7.9%, 35.9%)	7.0 (3.0, 17.8)
Etanercept	50.1% (35.2%, 65.5%)	3.0 (2.1, 5.4)	46.6% (26.6%, 71.2%)	2.5 (1.6, 5.1)	9.1% (2.7%, 32.6%)	15.2 (3.3, 318.5)
Golimumab	61.6% (45.9%, 76.3%)	2.2 (1.7, 3.4)	39.8% (22.0%, 64.7%)	3.1 (1.7, 6.6)	27.4% (9.2%, 71.8%)	4.0 (1.4, 14.6)
Infliximab	56.2% (39.9%, 72.2%)	2.6 (1.8, 4.3)	57.1% (32.7%, 82.5%)	2.0 (1.3, 3.9)	34.2% (12.1%, 77.9%)	3.2 (1.3, 10.3)
Secukinumab 150 mg	51.1% (414%, 61.0%)	2.9 (2.3, 4.0)	33.8% (23.5%, 46.8%)	3.7 (2.5, 6.0)	27.3% (13.4%, 53.1%)	4.0 (2.0, 9.0)
Secukinumab 300 mg	55.2% (41.0%, 68.8%)	2.6 (1.9, 4.1)	34.0% (20.6%, 51.0%)	3.7 (2.3, 7.2)	27.0% (11.3%, 55.7%)	4.1 (1.9, 11.1)
Ustekinumab 45 mg	35.4% (27.5%, 44.4%)	5.4 (3.7, 9.2)	19.9% (13.0%, 29.9%)	7.7 (4.4, 15.8)	10.2% (4.8%, 21.9%)	13.0 (5.2, 39.7)
Ustekinumab 90 mg	39.9% (31.5%, 49.1%)	4.4 (3.2, 6.7)	23.5% (15.6%, 34.3%)	6.1 (3.7, 11.1)	12.1% (5.9%, 25.2%)	10.4 (4.4, 27.8)

*CrI* credible interval, *NNT* number needed to treat

Similar rankings of ACR20/50/70 responses and NNTs for the different TIMs were observed in the analysis among biologic-naive patients (Additional file 4: Table S3). Biologic-naive patients treated with golimumab, adalimumab, secukinumab, or infliximab had higher ACR20 responses compared with other TIMs. Infliximab, etanercept, adalimumab, and golimumab had numerically higher ACR50 responses compared with other TIMs. In terms of ACR70, adalimumab, infliximab, golimumab, and secukinumab had higher efficacy than other TIMs among the biologic-naïve PsA population.

### Network meta-analysis: PASI outcomes

PsA patients treated with infliximab had the highest PASI75 responses at Week 24 (77.1%), followed by golimumab (74.1%), adalimumab (72.7%), and secukinumab 300 mg (60.4%). In terms of PASI90, infliximab had the highest efficacy compared with other TIMs (61.0%), followed by golimumab (57.2%), adalimumab (55.5%), and secukinumab 300 mg (42.3%). Detailed results of the NMA of PASI75 and PASI90 for all TIMs among the overall PsA population are shown in Table 2.

**Table 2:** PASI response rates and NNT at Week 24 among the overall population

Treatment	PASI75		PASI90	
	Response (95% CrI)	NNT (95% CrI)	Response (95% CrI)	NNT (95% CrI)
Placebo	7.6% (5.2%, 10.8%)	–	2.9% (1.8%, 4.5%)	–
Adalimumab	72.7% (54.0%, 86.7%)	1.5 (1.3, 2.1)	55.5% (36.0%, 74.3%)	1.9 (1.4, 3.0)
Apremilast	23.9% (14.1%, 36.5%)	6.2 (3.6, 13.3)	12.0% (6.1%, 21.0%)	11.0 (5.7, 26.5)
Certolizumab pegol	45.6% (31.6%, 60.4%)	2.6 (1.9, 4.0)	28.4% (17.4%, 42.1%)	3.9 (2.6, 6.7)
Etanercept	26.0% (12.9%, 44.1%)	5.5 (2.8, 16.7)	13.4% (5.5%, 27.1%)	9.6 (4.2, 34.1)
Golimumab	74.1% (56.1%, 87.7%)	1.5 (1.3, 2.0)	57.2% (37.9%, 75.8%)	1.8 (1.4, 2.8)
Infliximab	77.1% (60.5%, 89.5%)	1.4 (1.2, 1.9)	61.0% (42.4%, 78.8%)	1.7 (1.3, 2.5)
Secukinumab 150 mg	50.3% (36.1%, 65.3%)	2.3 (1.8, 3.4)	32.4% (20.6%, 47.2%)	3.4 (2.3, 5.5)
Secukinumab 300 mg	60.4% (39.7%, 79.2%)	1.9 (1.4, 3.0)	42.3% (23.6%, 63.7%)	2.5 (1.7, 4.8)
Ustekinumab 45 mg	51.2% (37.5%, 64.8%)	2.3 (1.8, 3.2)	33.2% (21.8%, 46.6%)	3.3 (2.3, 5.1)
Ustekinumab 90 mg	58.2% (44.7%, 70.8%)	2.0 (1.6, 2.6)	39.9% (27.5%, 53.4%)	2.7 (2.0, 4.0)

*CrI* credible interval, *NNT* number needed to treat

Similar rankings of TIMs in PASI75 and PASI90 responses and NNTs among biologic-naive patients were observed. Among biologic-naive patients, infliximab, golimumab, and adalimumab showed higher PASI75 and PASI90 responses than other TIMs while etanercept had lower efficacy in PASI outcomes. Detailed results among the biologic-naïve population are shown in Additional file 5: Table S4.

### Network meta-analysis: Incremental cost per responder over 24 weeks

**Fig. 2:**
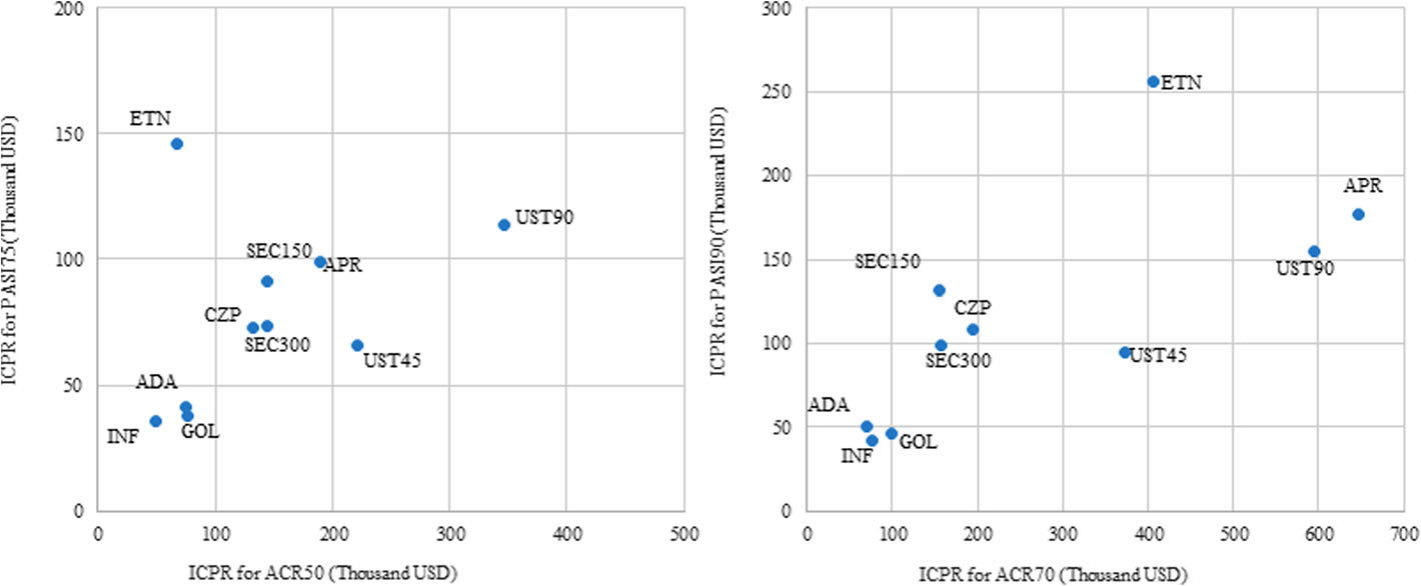
Incremental cost per responder over 24 weeks among the overall population. **a** Incremental cost per additional ACR50 responder vs. incremental cost per additional PASI75 responder. **b** Incremental cost per additional ACR70 responder vs. incremental cost per additional PASI90 responder. *ADA, adalimumab; APR, apremilast; CZP, certolizumab pegol; ETN, etanercept; GOL, golimumab; IFX, infliximab; SEC 150, secukinumab 150 mg; SEC 300, secukinumab 300 mg; UST 45, ustekinumab 45 mg; UST 90, ustekinumab 90 mg*

From a cost-effectiveness perspective, infliximab, adalimumab, and golimumab have demonstrated lower incremental cost per responder in both joint and skin outcomes compared to other TIMs. Evaluation of the cost-effectiveness over 24 weeks revealed that infliximab ($48,859 for ACR50/$35,277 for PASI75), adalimumab ($74,438 for ACR50/$41,013 for PASI75), and golimumab ($75,966 for ACR50/$37,542 for PASI75) were associated with the lowest incremental costs per additional ACR50 responder and per additional PASI75 responder (Fig. 2a). Similar conclusions can be reached using incremental cost per responder for ACR70 and PASI90. Adalimumab ($69,641 for ACR70/$50,717 for PASI90), infliximab ($77,347 for ACR70/$42,171 for PASI90), and golimumab ($100,158 for ACR70/$45,926 for PASI90) were associated with the lowest incremental costs per responder over 24 weeks (Fig. 2b).

A similar ranking of these TIMs in terms of cost-effectiveness was observed in the biologic-naïve population. Infliximab, adalimumab, and golimumab were consistently associated with lower incremental cost per responder over 24 weeks in both joint and skin outcomes compared to other TIMs (Additional file 6: Figure S2A and S2B). Detailed results of incremental costs per responder for all ACR and PASI outcomes among overall and biologic-naïve populations are shown in Additional files 7 and 8: Tables S5 and S6, respectively.

## Discussion

The treatment goal for PsA is to improve signs and symptoms of disease, including both peripheral arthritis (which is associated with radiographic progression and deterioration of physical function) and skin disease (which is associated with mental health and impaired quality of life) [26–28]. In this network meta-analysis, adalimumab, golimumab, and infliximab showed better efficacy and cost-effectiveness relative to other TIMs in both joint and skin outcomes. Specifically, these treatments had the lowest NNTs and incremental costs per additional responder over 24 weeks across all evaluated TIMs among the overall population as well as the biologic-naïve population. The rankings of other TIMs in terms of response rate, NNT, and incremental costs per additional responder were similar in the overall and biologic-naïve populations.

Several previous network meta-analyses / economic evaluations have investigated the efficacy and cost-effectiveness of TIMs for PsA. One conducted from the UK NHS perspective included adalimumab, etanercept, golimumab, and infliximab and concluded that etanercept was cost-effective – with similar joint responses by PsA response criteria (PsARC) despite lower PASI responses compared to adalimumab, golimumab, and infliximab [29, 30]. Another study, based on the same set of RCTs, concluded that golimumab and etanercept were associated with higher PsARC responses compared with adalimumab and infliximab [31]. Limitations of both analyses include inconsistent time points for outcome measurement. Thorlund et al. [31] used the last observed time point as the primary endpoint despite including some studies with only Week 12 endpoints [21, 23] and others with Week 24 endpoints. In the current systematic literature review, studies reporting only Week 12 outcomes were excluded from analyses to facilitate a fair comparison across TIMs. In addition, a third study (McInnes 2016) concluded comparable response rates between secukinumab and TNF inhibitors across all ACR outcomes in the mixed population of biologic-naïve and biologic-experienced PsA patients [32]. A recent meta-analysis conducted by Dongze et al. evaluated efficacy and safety of anti-cytokine biologic agents including secukinumab, ustekinumab, clazakizumab, and ixekizumab for active PsA at Week 24 and concluded that secukinumab and ustekinumab were the most efficacious short-term treatments among assessed agents [33]. Clazakizumab and ixekizumab were not included in this study since neither was approved as treatment for PsA at the time of the analysis.

Previous studies focused on PsARC as the measure of articular response, defined by ≥30% improvement in tender or swollen joint counts and one-point improvement in patient or physician global assessment of disease activity on a five-point Likert scale. In contrast, ACR response criteria also include visual analogue scale (VAS) scores of patient reported pain, Health Assessment Questionnaire (HAQ), and acute phase reactants. ACR responses are considered more stringent and comprehensive, and have become the primary outcome measure in a majority of RCTs in PsA [34]. Consequently, the current analysis chose ACR over PsARC responses as the measure of joint responses in PsA.

Biologic-naïve PsA patients generally report higher absolute joint and skin responses; however, comparative efficacy and cost-effectiveness in this population has not been systematically reviewed. Two recent meta-analysis studies comparing the efficacy of TIMs in biologic-naïve PsA patients concluded that ACR responses were higher among TNF inhibitors (etanercept, infliximab, adalimumab, and golimumab) and secukinumab, compared with other TIMs such as apremilast and ustekinumab [32, 35]. Another meta-analysis study conducted in a biologic-naïve PsA population in a Taiwanese setting found that etanercept had lower annual costs per PsARC and ACR20 responders than adalimumab and golimumab [36]. The above-mentioned studies only considered joint outcomes from Week 12 to Week 16, whereas the current study assessed efficacy in terms of both joint and skin outcomes at Week 24, as well as the cost-effectiveness of these TIMs. The longer time frame, inclusion of additional clinically relevant endpoints, and the cost analysis attest to the greater strength of the evidence presented in our study. The current analyses are in fact the first to include the more recently approved treatments (including ustekinumab, certolizumab pegol, secukinumab, and apremilast) and comprehensively evaluate their comparative efficacy and cost-effectiveness.

This network meta-analysis is subject to limitations commonly present in indirect comparison studies, despite synthesizing data from RCTs. Network meta-analyses may be biased due to the heterogeneity of patient populations across different trials. Although they adjust for placebo treatment responses which may account for trial-specific factors likely to influence outcomes in the active treatment arms, such adjustment only reduces the between-study heterogeneity. Moreover, due to the relatively small numbers of eligible RCTs for each pairwise comparison in this network meta-analysis, it was not possible to adjust for baseline risks within each trial. One specific limitation was that biologic-naïve data were not available for apremilast or certolizumab, and biologic-naïve results for secukinumab were based on a small sample size, which limited potential comparative interpretation. In addition, due to the lack of published data on patients who achieve both responses in terms of both joint and skin outcomes, the efficacy and cost-effectiveness of dual responses could not be assessed. However, treatment decisions should be based upon comprehensive considerations of both aspects. Despite the above limitations, this network meta-analysis represents the best evidence available to assess the comparative efficacy of currently available TIMs among PsA patients to inform health technology assessments and other decision-making [20, 37, 38].

## Conclusions

This study demonstrated the comparative efficacy and cost-effectiveness of TNF inhibitors, interleukin inhibitors, and phosphodiesterase-4 inhibitors for patients with active PsA in the US. Adalimumab, infliximab, and golimumab demonstrated better efficacy and lower incremental costs per responder by both joint and skin responses across all PsA patients as well as biologic-naive PsA patients. In the absence of comparative evidence from head-to-head trials, these results can inform more cost-effective use of these TIMs.

## Additional files

Additional file 1: Table S1. Summary of results at Week 24 from included trials for the overall population. (DOCX 40 kb)

Additional file 2: Table S2. Summary of results at Week 24 from included trials among biologic-naïve population. (DOCX 36 kb)

Additional file 3: Figure S1A. Evidence network for ACR and PASI outcomes among biologic-naïve population **(A)** Eleven trials reported ACR responses at Week 24 were selected: ADEPT [34], PALACE 1 [39], PALACE 4 [44, 45], RAPID-PsA [46], Mease 2004 [25], GO-REVEAL [47], IMPACT 2 [48], FUTURE 1 [49], FUTURE 2 [50], PSUMMIT 1 [51], and PSUMMIT 2 [52]. (B) Seven trials reported PASI responses at Week 24 were selected: ADEPT [34], Mease 2004 [25], GO-REVEAL [47], IMPACT 2 [48], FUTURE 2 [50], PSUMMIT 1 [51], and PSUMMIT 2 [52]. (TIFF 352 kb)

Additional file 4: Table S3. ACR response rates and NNT at Week 24 among biologic-naïve population. (DOCX 13 kb)

Additional file 5: Table S4. PASI response rates and NNT at Week 24 among biologic-naïve population^1^. (DOCX 12 kb)

Additional file 6: Figure S2. Incremental cost per responder over 24 weeks among the biologic-naïve population. **(A)** Incremental cost per additional ACR50 responder vs. Incremental cost per additional PASI75 responder. **(B)** Incremental cost per additional ACR70 responder vs. Incremental cost per additional PASI90 responder. PASI responses were not reported for certolizumab pegol or apremilast in the biologic-naïve population. *ADA, adalimumab; ETN, etanercept; GOL, golimumab; IFX, infliximab; SEC 150, secukinumab 150 mg; SEC 300, secukinumab 300 mg; UST 45, ustekinumab 45 mg; UST 90, ustekinumab 90 mg. (TIFF 105 kb)*

Additional file 7: Table S5. Incremental cost per responder over 24 weeks among the overall PsA population. (DOCX 13 kb)

Additional file 8: Table S6. Incremental cost per responder over 24 weeks among biologic-naïve population. (DOCX 13 kb)
