# Supplementary material for: Network meta-analysis and cost per responder of targeted Immunomodulators in the treatment of active psoriatic arthritis
Source: BMC Rheumatol. 2018 Feb 12;2:3. doi: 10.1186/s41927-018-0011-1 (PMC6390550; doi:10.1186/s41927-018-0011-1)
Supplement: Supplementary file 1 — Summary of results at Week 24 from included trials for the overall population. (DOCX 40 kb) [file 41927_2018_11_MOESM1_ESM.docx]

**Supplementary Table 1. Summary of results at Week 24 from included trials for the overall population**

|  | |  |  |  |  |  |  |  |  |  |
| --- | --- | --- | --- | --- | --- | --- | --- | --- | --- | --- |
| **Treatment** | **Arm** | **ACR20** | **ACR50** | **ACR70** | **PASI75** | **PASI90** | **Conventional DMARDs use at baseline** | | **MTX use at baseline** | |
| ADEPT[34] | Placebo | 24/162 | 10/162 | 2/162 | 1/69 | 0/69 | NR | | 81/162 | |
|  | Adalimumab 40mg EOW | 86/151 | 59/151 | 35/151 | 41/69 | 29/69 | NR | | 77/151 | |
| PALACE 1[39] | Placebo | 22/168 | 7/168 | 1/168 | 3/68 | NR | 110/168 | | 90/168 | |
|  | Apremilast 30mg BID | 59/168 | 32/168 | 17/168 | 17/82 | NR | 106/168 | | 88/168 | |
| PALACE 2[40] | Placebo | 25/159 | 14/159 | 5/159 | NR | NR | 113/159 | | 94/159 | |
|  | Apremilast 30mg BID | 40/162 | 19/162 | 4/162 | NR | NR | 113/162 | | 91/162 | |
| PALACE 3[41-43] | Placebo | 26/169 | 13/169 | 6/169 | 10/89 | NR | 101/169 | | 91/169 | |
|  | Apremilast 30mg BID | 52/167 | 27/167 | 9/167 | 23/90 | NR | 101/167 | | 83/167 | |
| PALACE 4[44, 45] | Placebo | 23/176 | 11/176 | 7/176 | NR | NR | 0/176 | | 0/176 | |
|  | Apremilast 30mg BID | 43/176 | 22/176 | 8/176 | NR | NR | 0/176 | | 0/176 | |
| RAPID-PsA[46] | Placebo | 32/136 | 17/136 | 6/136 | 13/86 | 5/86 | 88/136 | | 84/136 | |
|  | Certolizumab pegol 200mg EOW or 400mg Q4W | 164/273 | 115/273 | 71/273 | 102/166 | 69/166 | 199/273 | | 176/273 | |
| Mease 2004[25] | Placebo | 22/104 | 6/104 | 3/104 | 2/62 | 2/62 | NR | | 43/104 | |
|  | Etanercept 25mg BIW | 57/101 | 41/101 | 10/101 | 15/66 | 4/66 | NR | | 42/101 | |
| GO-REVEAL[47] | Placebo | 14/113 | 6/113 | 2/113 | 1/73 | 0/73 | NR | | 54/113 | |
|  | Golimumab 50mg Q4W | 76/146 | 47/146 | 28/146 | 57/102 | 33/102 | NR | | 71/146 | |
| IMPACT 2[48] | Placebo | 16/100 | 4/100 | 2/100 | 1/87 | 0/87 | NR | | 45/100 | |
|  | Infliximab 5mg/kg | 54/100 | 41/100 | 27/100 | 50/83 | 32/83 | NR | | 47/100 | |
| FUTURE 1[49] | Placebo | 35/202 | 15/202 | 4/202 | 9/109 | 4/109 | NR | | 125/202 | |
|  | Secukinumab 150mg Q4W | 101/202 | 70/202 | 38/202 | 66/108 | 49/108 | NR | | 121/202 | |
| FUTURE 2[50] | Placebo | 15/98 | 7/98 | 1/98 | 7/43 | 4/43 | NR | | 50/98 | |
|  | Secukinumab 300mg Q4W | 54/100 | 35/100 | 20/100 | 26/41 | 20/41 | NR | | 44/100 | |
|  | Secukinumab 150mg Q4W | 51/100 | 35/100 | 21/100 | 28/58 | 19/58 | NR | | 44/100 | |
| PSUMMIT 1[51] | Placebo | 47/206 | 18/206 | 5/206 | 16/146 | NR | NR | | 96/206 | |
|  | Ustekinumab 45mg Q12W | 87/205 | 51/205 | 25/205 | 83/145 | NR | NR | | 99/205 | |
|  | Ustekinumab 90mg Q12W | 101/204 | 57/204 | 29/204 | 93/149 | NR | NR | | 101/204 | |
| PSUMMIT 2[52] | Placebo | 21/104 | 7/104 | 3/104 | 4/80 | 3/80 | NR | | 49/104 | |
|  | Ustekinumab 45mg Q12W | 45/103 | 18/103 | 7/103 | 41/80 | 24/80 | NR | | 54/103 | |
|  | Ustekinumab 90mg Q12W | 46/105 | 24/105 | 9/105 | 45/81 | 36/81 | NR | | 52/105 | |

*BID, twice daily; BIW, twice weekly; DMARDs, disease-modifying antirheumatic drugs; EOW, every other week; MTX, methotrexate; Q4W, every 4 weeks; Q12W, every 12 weeks; NR, not reported.*
