# Supplementary material for: Network meta-analysis and cost per responder of targeted Immunomodulators in the treatment of active psoriatic arthritis
Source: BMC Rheumatol. 2018 Feb 12;2:3. doi: 10.1186/s41927-018-0011-1 (PMC6390550; doi:10.1186/s41927-018-0011-1)
Supplement: Supplementary file 2 — Summary of results at Week 24 from included trials among biologic-naïve population. (DOCX 36 kb) [file 41927_2018_11_MOESM2_ESM.docx]

**Supplementary Table 2. Summary of results at Week 24 from included trials among biologic-naïve population**

| **Treatment** | **Arm** | **ACR20** | **ACR50** | **ACR70** | **PASI75** | **PASI90** |
| --- | --- | --- | --- | --- | --- | --- |
| ADEPT[34] | Placebo | 24/162 | 10/162 | 2/162 | 1/69 | 0/69 |
|  | Adalimumab 40mg EOW | 86/151 | 59/151 | 35/151 | 41/69 | 29/69 |
| PALACE 1[39] | Placebo | 28/118 | NR | NR | NR | NR |
|  | Apremilast 30mg BID | 52/120 | NR | NR | NR | NR |
| PALACE 2[40] | Placebo | NR | NR | NR | NR | NR |
|  | Apremilast 30mg BID | NR | NR | NR | NR | NR |
| PALACE 3[41-43] | Placebo | NR | NR | NR | NR | NR |
|  | Apremilast 30mg BID | NR | NR | NR | NR | NR |
| PALACE 4[44, 45] | Placebo | 23/176 | 11/176 | 7/176 | NR | NR |
|  | Apremilast 30mg BID | 43/176 | 22/176 | 8/176 | NR | NR |
| RAPID-PsA[46] | Placebo | 29/110 | 16/110 | 5/110 | NR | NR |
|  | Certolizumab pegol 200mg EOW or 400mg E4W ^A^ | 132/219 | 91/219 | 57/219 | NR | NR |
| Mease 2004[25] | Placebo | 22/104 | 6/104 | 3/104 | 2/62 | 2/62 |
|  | Etanercept 25mg BIW | 57/101 | 41/101 | 10/101 | 15/66 | 4/66 |
| GO-REVEAL[47] | Placebo | 14/113 | 6/113 | 2/113 | 1/73 | 0/73 |
|  | Golimumab 50mg E4W | 76/146 | 47/146 | 28/146 | 57/102 | 33/102 |
| IMPACT 2[48] | Placebo | 16/100 | 4/100 | 2/100 | 1/87 | 0/87 |
|  | Infliximab 5mg/kg | 54/100 | 41/100 | 27/100 | 50/83 | 32/83 |
| FUTURE 1[49] | Placebo | 25/143 | 12/143 | 4/143 | NR | NR |
|  | Secukinumab 150mg E4W | 78/143 | 57/143 | 32/143 | NR | NR |
| FUTURE 2[50] | Placebo | 10/63 | 4/63 | 1/63 | 6/31 | 3/31 |
|  | Secukinumab 300mg E4W | 39/67 | 26/67 | 15/67 | 19/30 | 16/30 |
|  | Secukinumab 150mg E4W | 40/63 | 28/63 | 17/63 | 20/36 | 14/36 |
| PSUMMIT 1[51] | Placebo | 47/206 | 18/206 | 5/206 | 16/146 | NR |
|  | Ustekinumab 45mg E12W | 87/205 | 51/205 | 25/205 | 83/145 | NR |
|  | Ustekinumab 90mg E12W | 101/204 | 57/204 | 29/204 | 93/149 | NR |
| PSUMMIT 2[52] | Placebo | 12/42 | NR | NR | 3/30 | NR |
|  | Ustekinumab 45mg E12W | 23/43 | NR | NR | 21/36 | NR |
|  | Ustekinumab 90mg E12W | 26/47 | NR | NR | 25/40 | NR |

*BID, twice daily; BIW, twice weekly; EOW, every other week; Q4W, every 4 weeks; Q12W, every 12 weeks; NR, not reported.*
