# Supplementary figures and images for: Network meta-analysis and cost per responder of targeted Immunomodulators in the treatment of active psoriatic arthritis
Source: BMC Rheumatol. 2018 Feb 12;2:3. doi: 10.1186/s41927-018-0011-1 (PMC6390550; doi:10.1186/s41927-018-0011-1)

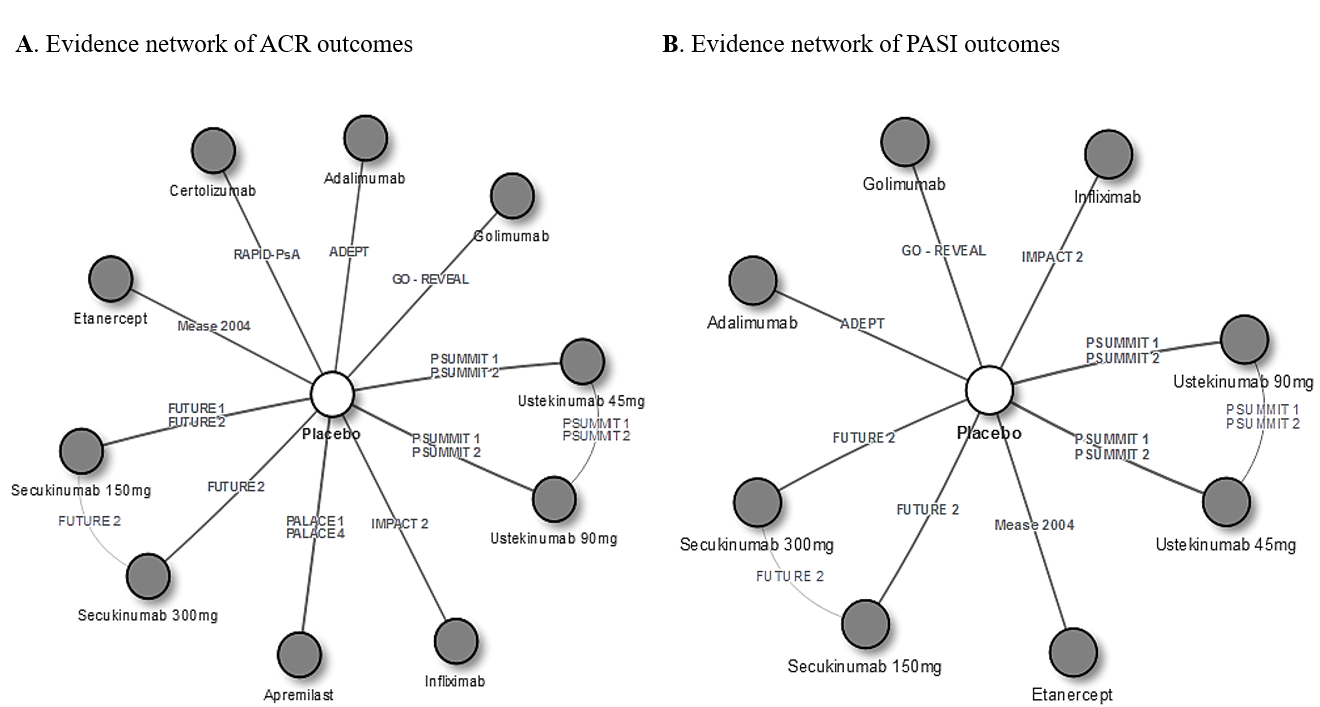

Supplement: Supplementary file 3 — Evidence network for ACR and PASI outcomes among biologic-naïve population (A) Eleven trials reported ACR responses at Week 24 were selected: ADEPT [34], PALACE 1 [39], PALACE 4 [44, 45], RAPID-PsA [46], Mease 2004 [25], GO-REVEAL [47], IMPACT 2 [48], FUTURE 1 [49], FUTURE 2 [50], PSUMMIT 1 [51], and PSUMMIT 2 [52]. (B) Seven trials reported PASI responses at Week 24 were selected: ADEPT [34], Mease 2004 [25], GO-REVEAL [47], IMPACT 2 [48], FUTURE 2 [50], PSUMMIT 1 [51], and PSUMMIT 2 [52]. (TIFF 352 kb) [file 41927_2018_11_MOESM3_ESM.tif]

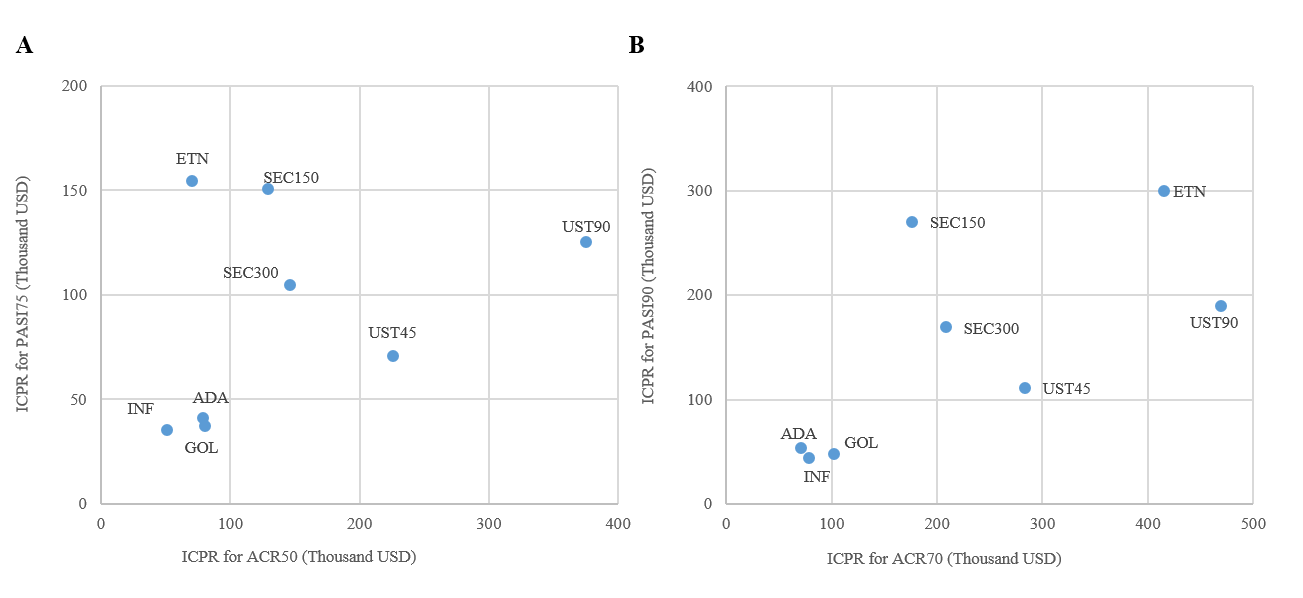

Supplement: Supplementary file 6 — Incremental cost per responder over 24 weeks among the biologic-naïve population. (A) Incremental cost per additional ACR50 responder vs. Incremental cost per additional PASI75 responder. (B) Incremental cost per additional ACR70 responder vs. Incremental cost per additional PASI90 responder. PASI responses were not reported for certolizumab pegol or apremilast in the biologic-naïve population. ADA, adalimumab; ETN, etanercept; GOL, golimumab; IFX, infliximab; SEC 150, secukinumab 150 mg; SEC 300, secukinumab 300 mg; UST 45, ustekinumab 45 mg; UST 90, ustekinumab 90 mg. (TIFF 105 kb) [file 41927_2018_11_MOESM6_ESM.tif]
