# Supplementary material for: Network meta-analysis and cost per responder of targeted Immunomodulators in the treatment of active psoriatic arthritis
Source: BMC Rheumatol. 2018 Feb 12;2:3. doi: 10.1186/s41927-018-0011-1 (PMC6390550; doi:10.1186/s41927-018-0011-1)
Supplement: Supplementary file 4 — ACR response rates and NNT at Week 24 among biologic-naïve population. (DOCX 13 kb) [file 41927_2018_11_MOESM4_ESM.docx]

**Supplementary Table 3. ACR response rates and NNT at Week 24 among biologic-naïve population**

| **Treatment** | **ACR20** | | **ACR50** | | **ACR70** | |
| --- | --- | --- | --- | --- | --- | --- |
|  | **Response rate**  **(95% CrI)** | **NNT**  **(95% CrI)** | **Response rate**  **(95% CrI)** | **NNT**  **(95% CrI)** | **Response rate**  **(95% CrI)** | **NNT**  **(95% CrI)** |
| Placebo | 17.5% (15.5%, 19.6%) | -- | 6.3% (5.0%, 7.8%) | -- | 2.4% (1.7%, 3.5%) | -- |
| Adalimumab | 62.0% (48.6%, 74.4%) | 2.2 (1.8, 3.2) | 40.2% (24.7%, 60.2%) | 2.9 (1.9, 5.3) | 40.2% (15.2%, 82.1%) | 2.6 (1.3, 7.7) |
| Apremilast | 32.9% (24.4%, 42.8%) | 6.5 (4.0, 13.6) | 12.7% (6.3%, 25.2%) | 15.6 (5.4, 558.7) | 2.8% (0.9%, 8.2%) | 290.7 (18.0, ∞) |
| Certolizumab pegol | 47.6% (35.2%, 60.8%) | 3.3 (2.3, 5.5) | 22.2% (13.3%, 35.9%) | 6.3 (3.4, 13.6) | 16.1% (6.9%, 37.8%) | 7.3 (2.9, 21.0) |
| Etanercept | 50.9% (35.8%, 66.5%) | 3.0 (2.1, 5.4) | 44.0% (24.4%, 69.2%) | 2.7 (1.6, 5.5) | 8.9% (2.5%, 32.6%) | 15.6 (3.3, 352.1) |
| Golimumab | 62.4% (46.6%, 77.0%) | 2.2 (1.7, 3.4) | 37.3% (20.0%, 62.6%) | 3.2 (1.8, 7.2) | 26.9% (8.8%, 71.6%) | 4.1 (1.4, 15.2) |
| Infliximab | 57.1% (40.6%, 72.9%) | 2.5 (1.8, 4.3) | 54.5% (30.2%, 81.2%) | 2.1 (1.3, 4.1) | 33.7% (11.5%, 77.8%) | 3.2 (1.3, 10.7) |
| Secukinumab 150mg | 58.5% (46.8%, 69.7%) | 2.4 (1.9, 3.4) | 36.4% (23.8%, 52.5%) | 3.3 (2.2, 5.6) | 24.4% (11.2%, 50.4%) | 4.5 (2.1, 11.1) |
| Secukinumab 300mg | 56.2% (39.1%, 72.3%) | 2.6 (1.8, 4.6) | 32.8% (17.7%, 53.4%) | 3.8 (2.1, 8.6) | 21.1% (7.8%, 49.5%) | 5.4 (2.1, 18.0) |
| Ustekinumab 45mg | 35.3% (26.6%, 45.2%) | 5.6 (3.7, 10.5) | 19.0% (11.3%, 31.0%) | 7.9 (4.1, 18.6) | 12.5% (5.0%, 31.6%) | 9.9 (3.5, 34.8) |
| Ustekinumab 90mg | 41.1% (31.8%, 51.3%) | 4.2 (3.0, 6.8) | 21.6% (13.1%, 34.4%) | 6.6 (3.6, 14.0) | 14.6% (6.1%, 35.4%) | 8.2 (3.1, 25.6) |

*CrI, credible interval; NNT, number needed to treat.*
