# Supplementary material for: Network meta-analysis and cost per responder of targeted Immunomodulators in the treatment of active psoriatic arthritis
Source: BMC Rheumatol. 2018 Feb 12;2:3. doi: 10.1186/s41927-018-0011-1 (PMC6390550; doi:10.1186/s41927-018-0011-1)
Supplement: Supplementary file 5 — PASI response rates and NNT at Week 24 among biologic-naïve population1. (DOCX 12 kb) [file 41927_2018_11_MOESM5_ESM.docx]

**Supplementary Table 4. PASI response rates and NNT at Week 24 among biologic-naïve population^1^**

| **Treatment** | **PASI75** | | **PASI90** | |
| --- | --- | --- | --- | --- |
|  | **Response (95% CrI)** | **NNT (95% CrI)** | **Response (95% CrI)** | **NNT (95% CrI)** |
| Placebo | 6.6% (4.3%, 9.8%) | -- | 2.2% (1.2%, 3.7%) | -- |
| Adalimumab | 71.7% (53.0%, 86.2%) | 1.5 (1.3, 2.1) | 52.2% (33.2%, 71.7%) | 2.0 (1.4, 3.2) |
| Etanercept | 23.9% (11.8%, 41.8%) | 5.8 (2.9, 17.8) | 11.1% (4.3%, 23.7%) | 11.2 (4.8, 41.0) |
| Golimumab | 73.3% (55.6%, 87.1%) | 1.5 (1.3, 2.0) | 54.3% (35.2%, 73.2%) | 1.9 (1.4, 3.0) |
| Infliximab | 76.3% (59.5%, 88.9%) | 1.4 (1.2, 1.9) | 57.9% (39.3%, 75.9%) | 1.8 (1.4, 2.7) |
| Secukinumab 150mg | 32.4% (13.7%, 59.2%) | 3.9 (1.9, 13.1) | 16.6% (5.4%, 39.2%) | 7.0 (2.7, 29.6) |
| Secukinumab 300mg | 43.8% (20.4%, 71.3%) | 2.7 (1.6, 6.9) | 25.2% (9.1%, 51.5%) | 4.4 (2.0, 13.9) |
| Ustekinumab 45mg | 47.1% (33.2%, 62.1%) | 2.5 (1.9, 3.6) | 28.0% (17.0%, 42.1%) | 3.9 (2.5, 6.5) |
| Ustekinumab 90mg | 52.3% (38.0%, 66.7%) | 2.2 (1.7, 3.1) | 32.4% (20.3%, 47.2%) | 3.3 (2.3, 5.4) |

[1] PASI75/90 responses are not reported for certolizumab pegol or apremilast in the biologic-naïve population.

*CrI, credible interval; NNT, number needed to treat.*
