# Supplementary material for: Network meta-analysis and cost per responder of targeted Immunomodulators in the treatment of active psoriatic arthritis
Source: BMC Rheumatol. 2018 Feb 12;2:3. doi: 10.1186/s41927-018-0011-1 (PMC6390550; doi:10.1186/s41927-018-0011-1)
Supplement: Supplementary file 7 — Incremental cost per responder over 24 weeks among the overall PsA population. (DOCX 13 kb) [file 41927_2018_11_MOESM7_ESM.docx]

**Supplementary Table 5. Incremental cost per responder over 24 weeks among the overall PsA population**

| **Treatment** | **ACR20**  **(95% CrI)** | **ACR50**  **(95% CrI)** | **ACR70**  **(95% CrI)** | **PASI75**  **(95% CrI)** | **PASI90**  **(95% CrI)** |
| --- | --- | --- | --- | --- | --- |
| Placebo | -- | -- | -- | -- | -- |
| Adalimumab | $60302  ($47272, $85738) | $74438  ($48210, $131670) | $69641  ($33471, $196399) | $41013  ($34008, $56249) | $50717  ($37555, $79533) |
| Apremilast | $97978  ($70119, $151647) | $189045  ($111453, $376978) | $646075  ($253625, $3569961) | $99141  ($58499, $213466) | $176990  ($91841, $424725) |
| Certolizumab pegol | $83080  ($61372, $125736) | $131977  ($79872, $245150) | $193922  ($82902, $490911) | $72708  ($53653, $109569) | $108628  ($71676, $183898) |
| Etanercept | $80641  ($55134, $145243) | $67211  ($41631, $134756) | $405662  ($88615, $8487898) | $145783  ($74989, $446174) | $255863  ($112589, $909035) |
| Golimumab | $55860  ($42143, $85399) | $75966  ($43286, $163863) | $100158  ($36017, $364335) | $37542  ($31345, $50375) | $45926  ($34414, $70406) |
| Infliximab^1^ | $62446  ($44502, $106042) | $48859  ($32457, $94297) | $77347  ($32508, $251490) | $35277  ($30140, $45684) | $42171  ($32453, $61477) |
| Secukinumab 150mg | $113746  ($88834, $156797) | $144859  ($98142, $230809) | $156285  ($76897, $350154) | $90855  ($68890, $131360) | $131616  ($88801, $212543) |
| Secukinumab 300mg | $101428  ($75176, $159703) | $143583  ($88629, $278703) | $158147  ($73106, $429509) | $73449  ($54797, $118142) | $98812  ($64317, $185339) |
| Ustekinumab 45mg | $155436  ($106181, $263740) | $221365  ($126725, $453284) | $373087  ($149110, $1135548) | $65814  ($51449, $91405) | $94521  ($66701, $146561) |
| Ustekinumab 90mg | $249811  ($180484, $384880) | $346648  ($212228, $636968) | $595047  ($254159, $1589336) | $113411  ($92926, $149212) | $154864  ($115278, $227527) |

[1] Drug cost of infliximab is based on an 80kg adult.

*CrI, credible interval.*
