# Supplementary material for: Network meta-analysis and cost per responder of targeted Immunomodulators in the treatment of active psoriatic arthritis
Source: BMC Rheumatol. 2018 Feb 12;2:3. doi: 10.1186/s41927-018-0011-1 (PMC6390550; doi:10.1186/s41927-018-0011-1)
Supplement: Supplementary file 8 — Incremental cost per responder over 24 weeks among biologic-naïve population. (DOCX 13 kb) [file 41927_2018_11_MOESM8_ESM.docx]

**Supplementary Table 6. Incremental cost per responder over 24 weeks among biologic-naïve population**

| **Treatment** | **ACR20**  **(95% CrI)** | **ACR50**  **(95% CrI)** | **ACR70**  **(95% CrI)** | **PASI75**  **(95% CrI)** | **PASI90**  **(95% CrI)** |
| --- | --- | --- | --- | --- | --- |
| Placebo | -- | -- | -- | -- | -- |
| Adalimumab | $59855  ($47122, $84622) | $78608  ($49812, $142393) | $70548  ($33496, $204540) | $41093  ($33798, $56915) | $53327  ($38558, $85163) |
| Apremilast^1^ | $104288  ($64581, $217492) | $250338  ($86724, $8954819) | $4659629  ($288605, ∞) | NR | NR |
| Certolizumab pegol | $91844  ($64363, $153073) | $173601  ($94845, $375140) | $201998  ($78748, $579568) | NR | NR |
| Etanercept | $79725  ($54806, $143522) | $70798  ($42561, $145488) | $415399  ($89119, $9384507) | $154348  ($78329, $473672) | $299747  ($127937, $1093202) |
| Golimumab | $55462  ($42072, $84529) | $80533  ($44530, $178575) | $101942  ($36082, $378052) | $37443  ($31267, $50136) | $47925  ($35319, $74424) |
| Infliximab^2^ | $61847  ($44381, $104592) | $50812  ($32810, $101346) | $78467  ($32550, $262857) | $35227  ($29991, $45797) | $44052  ($33403, $65524) |
| Secukinumab 150mg | $94552  ($74872, $130340) | $128995  ($84788, $216759) | $176415  ($81467, $429176) | $150624  ($75056, $509883) | $269924  ($106332, $1147922) |
| Secukinumab 300mg | $100170  ($71083, $176656) | $146496  ($82979, $331561) | $208417  ($82830, $696410) | $104854  ($60835, $267687) | $169228  ($79262, $537141) |
| Ustekinumab 45mg | $161307  ($105166, $299956) | $225588  ($118311, $532684) | $283507  ($98948, $994995) | $70771  ($53040, $102609) | $111192  ($72882, $186800) |
| Ustekinumab 90mg | $242507  ($171815, $387748) | $374945  ($207473, $798766) | $469650  ($175213, $1465973) | $125450  ($97618, $175829) | $189205  ($129591, $308544) |

[1] 95% CrI for apremilast extends to infinity and include a region where placebo is more effective than apremilast.

[2] Drug cost of infliximab is based on an 80kg adult.

*CrI, credible interval. NR, Not Reported.*
